# Genetic disorders and male infertility

**DOI:** 10.1002/rmb2.12336

**Published:** 2020-06-27

**Authors:** Shinnosuke Kuroda, Kimitsugu Usui, Hiroyuki Sanjo, Teppei Takeshima, Takashi Kawahara, Hiroji Uemura, Yasushi Yumura

**Affiliations:** ^1^ Department of Urology, Reproductive Centre Yokohama City University Medical Centre Kanagawa Japan; ^2^ Department of Medical Genetics Yokohama City University Medical Centre Kanagawa Japan; ^3^ Department of Urology and Renal Transplantation Yokohama City University Medical Centre Kanagawa Japan

**Keywords:** azoospermia, genetic disorder, Klinefelter syndrome, male infertility, sex chromosome aberrations

## Abstract

**Background:**

At present, one out of six couples is infertile, and in 50% of cases, infertility is attributed to male infertility factors. Genetic abnormalities are found in 10%‐20% of patients showing severe spermatogenesis disorders, including non‐obstructive azoospermia.

**Methods:**

Literatures covering the relationship between male infertility and genetic disorders or chromosomal abnormalities were studied and summarized.

**Main findings (Results):**

Genetic disorders, including Klinefelter syndrome, balanced reciprocal translocation, Robertsonian translocation, structural abnormalities in Y chromosome, XX male, azoospermic factor (AZF) deletions, and congenital bilateral absence of vas deferens were summarized and discussed from a practical point of view. Among them, understanding on AZF deletions significantly changed owing to advanced elucidation of their pathogenesis. Due to its technical progress, AZF deletion test can reveal their delicate variations and predict the condition of spermatogenesis. Thirty‐nine candidate genes possibly responsible for azoospermia have been identified in the last 10 years owing to the advances in genome sequencing technologies.

**Conclusion:**

Genetic testing for chromosomes and AZF deletions should be examined in cases of severe oligozoospermia and azoospermia. Genetic counseling should be offered before and after genetic testing.

## 1 INTRODUCTION

Infertility is defined as the inability to become pregnant after at least 1 year of regular intercourse without contraception. It is reported that approximately 8%‐12% of couples in reproductive age are infertile, and male factors contribute to the infertility of those couples in approximately 50% of cases.^1^ Azoospermia has been reported in approximately 1% of all men and in 10%‐15% of infertile men.^2^ According to this percentage, male infertility and azoospermia could be currently considered a common disease. Nonetheless, the majority of male infertilities are categorized as idiopathic, representing about 50% of all cases, and their causes are unknown.^3^ Thus, one of the most important tasks for physicians and researchers engaged in reproductive medicine is to classify idiopathic male infertility based on its causes and reveal their pathogenic details. In cases of non‐obstructive azoospermia (NOA) and severe oligo‐astheno‐tetrazoospermia, chromosomal or genetic disorders were confirmed in 15%‐20% of patients.^4^ A recent review reported that genetic disorders could possibly explain at least some of these idiopathic cases.^5^

In fact, in clinical practice, Y chromosome microdeletion analysis has become routine for patients with severe oligozoospermia and azoospermia. Y chromosome microdeletions indicate genomic deletions in the region of azoospermic factor (AZF) spreading on the Y chromosome. The deletion of AZF is currently the only predictor of spermatogenic condition and success rate of micro‐testicular sperm extraction (micro‐TESE) in patients with azoospermia. In addition, owing to recent progress in genome‐analyzing technologies, especially in the last 10 years, studies have identified many genetic variations which associated with male infertility.^6^ Next‐generation sequencing technologies have made a particularly significant contribution to the search for candidate genes.^7^, ^8^ Moreover, not only genomic but also epigenetic mechanisms have been recently investigated.^9^, ^10^, ^11^ Epigenetics regulates gene expression and genome stability without altering DNA sequence via reversible modifications of chromatin in either DNA or histones and, in some cases, both DNA and histones.^9^

To date, genetic testing for chromosomal abnormalities and AZF deletions can provide important information to doctors and patients for decision making. However, no specific genes for any subgroup of “idiopathic” infertility have been identified and the exact relationships between genetics and impaired spermatogenesis remain mostly unclear. Nonetheless, the unveiling pathophysiology of male infertility through a genetic approach has a certain potential to contribute to an increased pregnancy rate in the era of artificial reproductive technology. In this review, we highlight literatures covering the relationship between male infertility and genetic disorders or chromosomal abnormalities.

## 2 TESTING FOR GENETIC ABNORMALITY IN CLINICAL PRACTICE

### 2.1 Chromosomal analysis

Chromosomal disorders are confirmed in 5% of patients with severe oligozoospermia and in 10%‐15% of patients with azoospermia.^12^, ^13^ Usually, a lymphocyte culture (72 hours) is performed to analyze the chromosomes. In routine analysis, 20 cells are analyzed. In cases of chromosomal mosaicism or chromosomal abnormalities, 30 cells are analyzed.^14^

Table 1 shows the chromosome abnormalities in individuals with male infertility. Klinefelter syndrome (KS) is the most common sex chromosome disorder responsible for male infertility.^15^ Its karyotype has two or more X chromosomes in males; 47,XXY is the most common karyotype. Symptoms are typically more severe if three or more X chromosomes are present (48,XXXY or 49,XXXXY).^16^ The prevalence of KS was reported to be approximately 1 in 1000 newborn males during the 1970s and 1980s.^17^, ^18^ In 1990, Danish registry studies described the prevalence of KS to be 153‐173 in every 100 000 newborn males.^19^ Recent studies reported that the prevalence was increasing, and 1 in 500‐600 newborn males had KS.^20^, ^21^, ^22^ Crawford stated that this change may be due to increasing awareness and optimization of diagnostic methods.^21^ Semen analysis of non‐mosaic KS patients usually shows azoospermia, while ejaculated spermatozoa are sometimes confirmed in patients with mosaic KS (46,XY/47,XXY). In cases of azoospermic KS, the sperm retrieval rate (SRR) with micro‐TESE was reported to be between 40% and 70%,^23^, ^24^, ^25^, ^26^, ^27^, ^28^ which was higher than those in unexplained NOA patients, that were reported to be between 31% and 42.9%.^28^, ^29^

**TABLE 1:** Clinical features of chromosomal abnormalities

	Typical karyotype	Frequency	Semen analysis	Treatment
Klinefelter syndrome	47,XXY	0.1%‐0.5% in male births	Azoospermia in most cases	Micro‐TESE—ICSI
Balanced reciprocal translocation	Various pattern	0.123% in whole population	Normozoospermia~Azoospermia	Depends on SA
Robertsonian translocation	45,XY,rob(14q15q)	0.9%‐3.4% in infertile men	Normozoospermia~Severe OAT	Depends on SA
Structural abnormality of Y chromosome	46,XY,del(Yq)	Unknown	Oligozoospermia~Azoospermia	Micro‐TESE—ICSI in case without AZF deletion
XX male	46,XX	0.005%‐0.001% in male births	Azoospermia	None

Abbreviations: AZF, azoospermic factor; ICSI, intracytoplasmic sperm injection; Micro‐TESE, micro‐dissection testicular sperm extraction; OAT, oligo‐astheno‐teratozoospermia; SA, semen analysis.

Chromosomal translocations are the most common structural disorders in men with a frequency of 1.23 per 1000,^30^ and their prevalence is 10 times greater in the infertile population.^31^ Chromosomal translocations are divided into balanced and unbalanced translocations. Balanced reciprocal translocation is an exchange of genetic material between two or more chromosomes. There are autosomal and sex chromosome translocations in balanced reciprocal translocations. Depending on the breakpoints, approximately 60% of the carriers of autosomal translocations have at least one abnormal parameter in their semen analysis.^32^ Although the frequency of sex chromosome translocations is rare, some reports have shown an association between Y chromosome translocations and azoospermia.^33^, ^34^, ^35^

Robertsonian translocation is the most common form of unbalanced chromosomal translocation in humans and is also the common cause of male infertility. Robertsonian translocations are found in 0.9%‐3.4% of infertile men with severe spermatogenic dysfunction.^36^, ^37^, ^38^ These can occur in five acrocentric chromosome pairs (13, 14, 15, 21, 22) and cause them to break at their centromeres, causing the two long arms to fuse together resulting in a single large chromosome. Thus, individuals with a Robertsonian translocation have 45 chromosomes. The remnants of the short arms of the two fused chromosomes are usually lost. Despite this genetic abnormality, carriers of Robertsonian translocation are phenotypically normal because the short arms of the two acrocentric chromosomes contain no important genes. However, the carriers are at increased risk of sperm aneuploidy, which could result in miscarriage or babies with translocated trisomy. Theoretically, one‐sixth of carriers' sperm have a normal karyotype, another one‐sixth carries Robertsonian translocation, and the remaining two thirds are in unbalanced states, in either nullisomy or disomy of chromosomes involved in translocations. However, the proportion of unbalanced sperm in ejaculated semen of Robertsonian translocation carriers is reported to be between 5.8% and 32%,^39^, ^40^, ^41^ which is much lower than the theoretical value due to natural selection during spermatogenesis. Nonetheless, male carriers of Robertsonian translocation have a higher rate of experiencing miscarriage or having babies with translocated trisomy. Scriven et al^42^ summarized the empirical data of common karyotypes of Robertsonian translocations; for female carriers of 45,XY,rob(14q21q), the estimated possibility of a translocated trisomy 21 prenatal diagnosis during the second trimester is 15%, while for male carriers, this possibility is <0.5%. 45,XY,rob(14q15q) may cause uniparental disomy (UPD). UPD is the inheritance of both homologous chromosomes from the same parent. UPD may cause abnormal phenotype through the effect of imprinting or non‐inheritance of recessive genes. Prader‐Willi syndrome and Angelman syndrome are known to be associated with maternal and paternal UPD of chromosome 15.^43^

Structural abnormalities in the Y chromosome that are responsible for male infertility include many variations, such as macro‐deletions of the long arm of the Y chromosome (del(Yq), ring Y) or duplication of the Y chromosome (dup(Y)).^44^ Those abnormal Y chromosomes are sometimes described as marker chromosomes (mar+) by regular chromosome analysis. Although these patients usually show azoospermia, micro‐TESE could be indicated under the condition that the AZF regions were not included in the deleted segment. Even when a Yq deletion harbors the AZF region, micro‐TESE could still be considered under the same indication as in the case of AZF microdeletion. However, in case that those Y chromosomes are supposed to be inherited to their male offspring, genetic counseling should be carefully provided.

The 46,XX male sex reversal syndrome was first reported in 1964 by de la Chapelle et al.^45^ It is one of the rarest sex chromosomal aberrations in male infertility. Guellaen et al^46^ reported in their study that the frequency of 46,XX male sex reversal syndrome is one in 20 000‐30 000 newborn males. Around 80% of these males consist of individuals with genital ambiguity, who have the sex‐determining region Y (*SRY*) gene on the X chromosome or autosomes, while 20% of XX males were *SRY‐*negative demonstrating higher incidence of genital ambiguity, hypospadias, cryptorchidism, and different degrees of masculinization.^47^, ^48^ All 46,XX males were totally infertile due to the lack of the AZF region in the long arm of the Y chromosome.^49^

### 2.2 Y chromosome microdeletion

**FIGURE 1:**
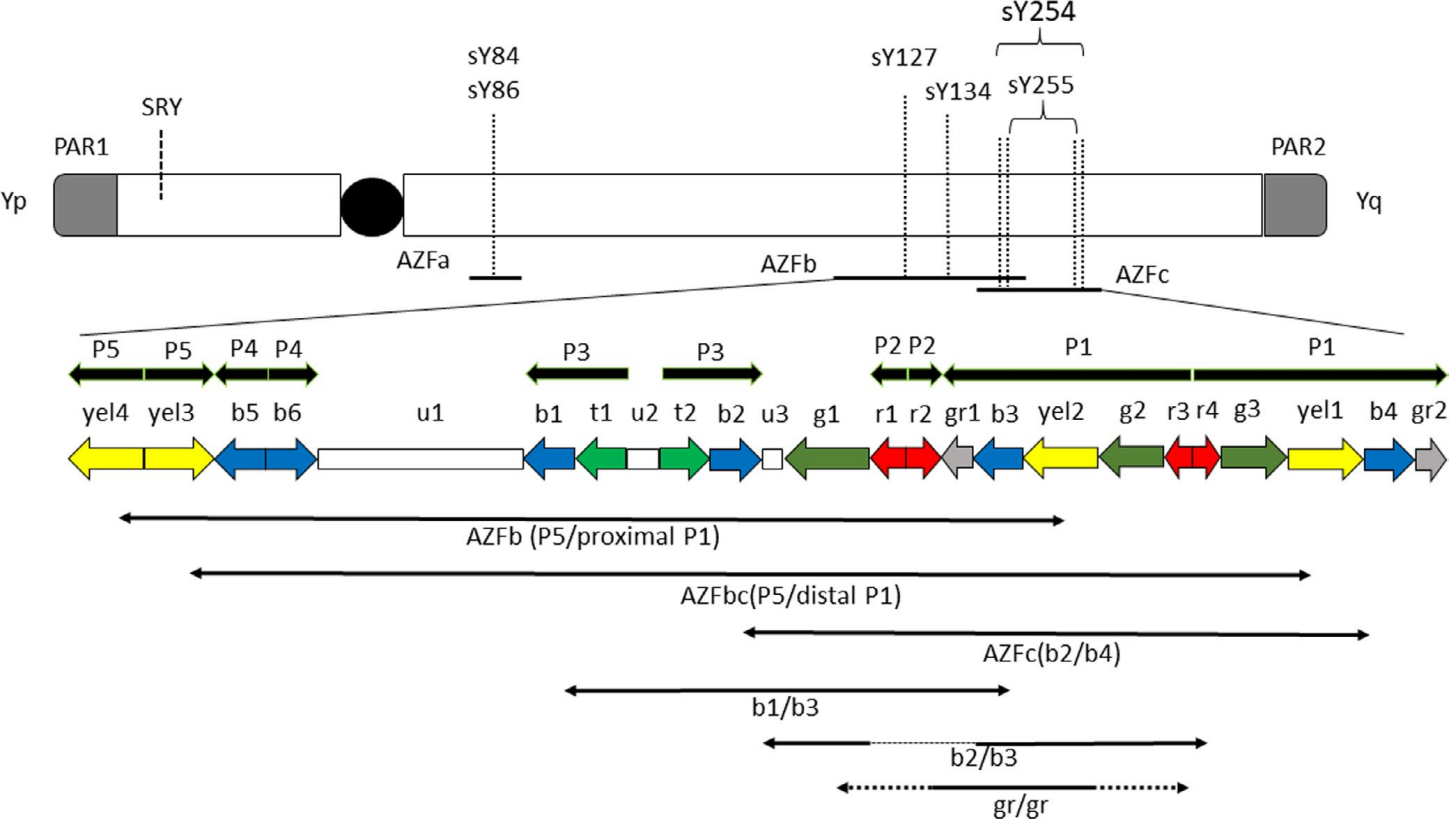
Scheme of deletion patterns of AZF region based on palindromes in Y chromosome long arm. The STS primers suggested by guidelines (sY85, sY86, sY127, sY134, sY254, sY255) are also located. In addition to AZF deletions (AZFa, AZFb, AZFb+c, AZFc), partial deletions (b1/b3, b2/b3, gr/gr) are indicated by arrows

Y chromosome microdeletions usually are deletions in the euchromatic part of the long arm of the Y chromosome, including AZF regions. AZF deletion is currently the only predictor of spermatogenic condition and contributes to the success rate of micro‐TESE in patients with azoospermia.^50^ AZF was classically subdivided into AZFa, AZFb, and AZFc when the Y chromosome sequence was not completely revealed.^51^ Vogt et al studied a large number of male patients and divided AZF into a, b, and c using molecular mapping of the male‐specific region of the Y chromosome (MSY) along with histological findings of the testis. After the completion of MSY's physical map and genomic sequencing in 2003, the ampliconic sequences were found to have more than 99% identity and to be organized in massive palindromes. Palindrome sequences thus showed a nearly complete symmetry, which enables them to form a hairpin loop. This structure enables the Y chromosome to conduct homologous recombination, by which DNA repair can be done even in the absence of a corresponding homologous chromosome, which autosomes usually utilize. This mechanism is considered important to maintain the function and diversity in MSY.^52^ Based on the palindrome structure, a detailed model of deletions is proposed (Figure 1).^53^ AZFb (P5/proximal P1) and AZFc (b2/b4) regions are partly overlapping. For the detection of AZFa, AZFb, and AZFc, PCR primers should at least include sY14(*SRY*), ZFX/ZFY, sY84, sY86, sY127, sY134, sY254, and sY255.^50^ Attention must be paid to whether different primers were used by researchers for the diagnosis of AZF deletions.

The AZFa region spans 1100 kb and contains only two genes, *USP9Y* and *DDX3Y*. This region also contains retroviral sequences such as HERVyq1 and HERVyq2 that have been acquired in humans through the evolutionary process. Between these same directional retroviral sequences which are flanking AZFa, homologous recombination could occur resulting in the deletion of AZFa.^54^, ^55^, ^56^

The AZFb region spans 6.2 Mb and contains 32 gene copies and transcription units including HSFY and RPS4Y2.^53^ Again, based on the palindrome structure, the AZFb deletion is located between P5 and proximal P1, which is supposed to occur by homologous recombination between the palindromes.^57^ The histological phenotype in patients with complete AZFb or AZFb+c deletions shows Sertoli cell only syndrome or maturation arrest, both leading to azoospermia.^50^, ^58^, ^59^

The AZFc region spans 3.5 Mb which contains 12 genes and transcription units including DAZ, GOLGA2LY, and CSPG4LY. Complete AZFc deletion arises by homologous recombination between amplicon b2 and b4. This condition presents variable degrees of spermatogenic impairment.^60^, ^61^ Generally, AZFc deletion induces hypo‐spermatogenesis, which often appears as azoospermia, severe oligozoospermia, or is revealed by histological examination. Zhao et al^62^ reported 183 cases with AZFc deletion in China; 105 patients had ejaculated sperm and 6 achieved natural pregnancy. This study suggests that AZFc deletion presents with variable levels of spermatozoal generation, even more than we expected.

In clinical practice, indication for surgical micro‐TESE for azoospermic patients must be considered upon test results of deletions in AZFa, AZFb, and AZFc.^50^ In 2003, Hopps et al^58^ reported a very poor SRR (0%) in patients having deletion of AZFa or AZFb, and a high SRR (75%) in those with AZFc deletion. According to the latest recommendations from American Society of Reproductive Medicine, sperm retrieval is hopeless in patients with AZFa, AZFb, and AZFb+c deletions. On the other hand, micro‐TESE could be recommended for the patients with AZFc deletion, although it should be well informed that the deletion will be inherited almost certainly to their sons.^63^ Pregnancy and live birth rates with intracytoplasmic sperm injection in patients having AZFc deletions were reported to be comparable to those in patients without deletions,^64^, ^65^ whereas some studies reported decreased outcomes of fertilization rate and embryo quality, or lower chance of pregnancy after intracytoplasmic sperm injection.^66^, ^67^, ^68^

### 2.3 AZF‐partial deletions

As the palindrome structures in the Y chromosome have been revealed, the existence of partial deletions in the AZF region was clarified by many studies. For instance, innovative diagnostic kits using precise sequence‐tagged‐site markers were developed and have provided new data on partial deletions in the AZF region.^69^ However, the influence of these partial deletions on spermatogenesis is still unclear. Thus, partial deletions are not the factors that definitively favor for or against the use of artificial reproductive technology or micro‐TESE for these patients. Again, it should be remembered that the deletions will be inherited to their sons.

Gr/gr deletions that involve the removal of the 1.6 Mb segment, nearly half of the AZFc region, form a category of AZFc deletion caused by the recombination between amplicons g1/g2, r1/r3, and r2/r4. It includes one copy of the CDY1 (CDY1a) gene, two copies of the DAZ (DAZ1/DAZ2) gene, and one copy of the BPY2 gene.^70^, ^71^ Other combinations of deletions were also reported: DAZ1/DAZ2+CDY1a, DAZ1/DAZ2+CDY1b, DAZ3/DAZ4+CDY1a, and DAZ3/DAZ4+CDY1b.^72^ The effect of these deletions on patient fertility largely depends on the ethnic and geographic origin of the population. There are a lot of reports that showed adverse effects of gr/gr deletions in spermatogenesis, especially in Caucasian populations.^73^, ^74^, ^75^ On the other hand, no negative effects on spermatogenesis have been reported in Asian population studies, including Japan and China.^76^, ^77^ The reason for these differences is still unclear but may be attributed to Y haplogroups and deletion subtypes. In the Japanese population, it has been reported that gr/gr deletions were found in 33.7% (260/772) of all cases examined, and the deletions were widespread in haplogroup D of the Y chromosome (86.2%). This indicated that gr/gr deletions do not influence spermatogenesis in the Japanese population.^77^ In 2019, Iijima et al,^78^ the same research group, reported almost the same proportion of gr/gr deletions among 1030 infertile males in Japan. However, they also stated that SRR in patients with gr/gr deletion was relatively lower than that in patients without the deletion (18.8% vs 28.7%, *P* = .09), although the difference was not statistically significant. Therefore, its clinical significance is still controversial.

The b2/b3 deletion removes 1.8 Mb of the AZFc section. The mechanism of b2/b3 deletion is complicated, the b2/b3 or gr/rg deletion is followed by a gr/rg or b2/b3 inversion.^79^, ^80^ Among the Chinese population, the association of b2/b3 partial deletion with male infertility was reported in 2009.^81^ On the contrary, studies in other populations did not show any association with infertility.^82^, ^83^ Yuan et al reported the natural transmission of b2/b3 sub‐deletion. They performed Y microdeletion tests for each father of four infertile male patients with complete deletions of AZFc or AZFb+c. The b2/b3 sub‐deletions were found in all fathers, though the fathers are not infertile, and the sons were all born through natural delivery.^84^

The b1/b3 deletion removes 1.6 Mb of the AZFc region. This deletion was defined as the loss of sY1161, sY1191, and sY1291 with the presence of other sequence‐tagged sites. The mechanism of b1/b3 deletion involves homologous recombination, possibly between sister chromatids or within a chromatid.^85^ Its frequency varies in previous reports.^86^, ^87^, ^88^, ^89^ Due to its low frequency, the effects of b1/b3 deletion on spermatogenesis remain unclear.^80^

### 2.4 Congenital bilateral absence of vas deferens

Congenital bilateral absence of vas deferens (CBAVD) is one of the causes of obstructive azoospermia. It is sometimes observed as a symptom of cystic fibrosis, a genetic condition causing exocrine gland disorders. Cystic fibrosis and isolated CBAVD are autosomal recessive and are recognized as cystic fibrosis transmembrane conductance regulator (CFTR)‐related diseases.^90^ Yu et al^91^ reported in their meta‐analysis that 78% of patients with CBAVD had at least one CFTR mutation, and the 5T allele and 5T/(TG)12_13 may contribute to the increased risk of CBAVD. In Japan, due to the very low frequency of CFTR mutation, commercial‐based tests for this mutation are not available.

### 2.5 New candidate genes in male infertility

As described above, the AZF region of the Y chromosome contains genes that affect spermatogenesis. Genome‐wide association studies during the past ten years^92^, ^93^, ^94^, ^95^, ^96^, ^97^, ^98^, ^99^, ^100^, ^101^, ^102^, ^103^, ^104^, ^105^, ^106^, ^107^, ^108^, ^109^, ^110^, ^111^, ^112^, ^113^, ^114^, ^115^, ^116^, ^117^, ^118^, ^119^, ^120^, ^121^ have brought significant improvement in genetic analysis techniques and many autosomal and X‐chromosomal genes have been reported to be possibility associated with spermatogenetic disorders. Representative candidate genes reported to be associated with oligozoospermia, asthenozoospermia, and azoospermia are listed in Table 2 Currently, there is no gene mutation or deletion definitively associated with male infertility as those observed in the AZF region. However, there are several genes that could be potential candidate markers for male infertility. For example, the TEX11 gene on the X chromosome (Xq13.2) is reported to play a key role in human meiosis. It encodes a 104 kDa protein in vertebrates and is considered a meiosis‐specific factor which is involved in double‐strand DNA break repair. Histological analysis showed maturation arrest in azoospermic men with *TEX11* mutations.^92^ Especially in idiopathic NOA or severe oligozoospermia patients, a broad diagnostic panel of genes would help to reach more accurate diagnoses.^122^

**TABLE 2:** The identified genes located in autosomes and X chromosome possibly implicated in male infertility

Phenotype	Gene	OMIM number	Location	Reference	Year
Asthenozoospermia	*CATSPER1*	606389	11q13.1	Avenarius et al^106^	2009
	*DNAAF2*	612517	14q21.3	Ji et al^110^	2017
	*DNAH5*	603335	5p15.2	Ji et al^110^	2017
	*DNAI1*	604366	9p13‐p21	Ji et al^110^	2017
	*GALNTL5*	615133	7q36.1	Takasaki et al^107^	2014
	*DYX1C1*	608706	15q21.3	Ji et al^110^	2017
	*SLC26A8*	608480	6p21.31	Dirami et al^108^	2013
	*HYDIN*	610812	16q22.2	Ji et al^110^	2017
	*SPAG17*	616554	1p12	Xu et al^109^	2018
	*LRRC6*	614930	8q24.22	Ji et al^110^	2017
Oligozoospermia/OAT/Azoospermia	*CCDC39*	613798	3q26.33	Ji et al^110^	2017
	*DAX1*	300473	Xp21.2	Mou et al^93^	2015
	*MAGEB4*	300153	Xp21.2	Okutman et al^95^	2017
	*TAF4B*	601689	18q11.2	Ayhan et al^96^	2014
	*HSF2*	140581	6q22.31	Mou et al^97^	2013
	*KLHL10*	608778	17q21.2	Yatsenko et al^98^	2006
	*TDRD6*	611200	6p12.3	Sha et al^111^	2018
	*HIWI*	605571	12q24.33	Gou et al^100^	2017
	*SPINK2*	605753	4q12	Kherraf et al^101^	2017
	*NANOS1*	608226	10q26.11	Kusz‐Zamelczyk et al^105^	2013
	*HAUS7*	300540	Xq28	Li et al^112^	2018
	*SEPT12*	611562	16p13.3	Kuo et al^113^	2012
NOA	*DNAH6*	603336	2p11.2	Gershoni et al^99^	2017
	*DMC1*	602721	22q13.1	He et al^114^	2018
	*DMRT1*	602424	9p24.3	Lopes et al^115^	2013
	*TEX11*	300311	Xq13.1	Yatsenko et al^92^	2015
	*TEX14*	605792	17q22	Gershoni et al^99^	2017
	*TEX15*	605795	8q12	Okutman et al^94^	2015
	*SOHLH1*	610224	9q34.3	Choi et al^102^	2010
	*NPAS2*	603347	2q11.2	Ramasamy et al^103^	2015
	*TDRD9*	617963	14q32.33	Arafat et al^104^	2017
	*FANCM*	609644	14q21.2	Kasak et al^116^	2018
	*MEIOB*	617670	16p13.3	Gershoni et al^99^	2017
	*NR5A1*	184757	9p33.3	Bashamboo et al^117^	2010
	*PLK‐4*	605031	4q28.1	Miyamoto et al^118^	2016
	*SYCE1*	611486	10q26.3	Maor‐Sagie et al^119^	2015
	*SYCP3*	604754	12q23.2	Stouffs et al^120^	2005
	*USP26*	300309	Xq26.2	Ma et al^121^	2016
	*ZMYND15*	614312	17p13.2	Ayhan et al^96^	2014

Abbreviations: NOA, non‐obstructive azoospermia; OAT, oligo‐astheno‐teratozoospermia; OMIM, online Mendelian inheritance in man.

### 2.6 Genetic counseling

To every couple who receives genetic tests, genetic counseling is mandatory to provide information on the disorder, treatment options for infertility, and information on the probability of conceiving babies having chromosomal or genetic disorders. Ideally, pre‐test counseling should also be offered to patients to improve understanding of the merits and demerits of the test. Most of azoospermic patients with chromosomal abnormalities, other than XX male and some cases of Y chromosome macro‐deletion, are eligible for micro‐TESE. The inheriting rate of those chromosomal anomalies to the next generation depends on the type of anomaly and is a current subject of much discussion. In cases with AZF deletions, the complete deletion of AZFa, AZFb, AZFbc, or AZFabc indicates that sperm production is zero. Thus, micro‐TESE should not be recommended. As for AZFc deletions, couples should realize that the deletion may be transmitted to the son with high possibility, although the SRR is relatively higher than in cases with unexplained NOA. In addition, the exact testicular phenotype of the son cannot be predicted because the AZFc phenotype varies in each individual due to different genetic backgrounds and environmental factors.

## 3 CONCLUSIONS AND FUTURE PERSPECTIVES

Chromosomal analysis and testing for AZF deletions should be performed in cases of severe oligozoospermia and azoospermia. Especially in cases of azoospermia, these examinations are mandatory to consider the indication for micro‐TESE. Except AZF deletions, there are no other currently available genetic markers for male infertility or for predicting the success rate of sperm retrieval in azoospermic patients, although many candidate genes that may be responsible for azoospermia have been identified over the last 10 years. Although we should recognize multifactorial aspects and genetic heterogeneity of male infertility, the potential to better define male infertility may increase in the next decade due to the advances in next‐generation sequencing. Genetic counseling should be offered in pre‐ and post‐chromosome and genetic mutation analysis.

## DISCLOSURES

*Conflict of interest*: The authors report no conflicts of interest. *Human/Animal rights statement*: This article does not contain any studies with human or animal subjects.
